# Learning Force Field Parameters from Differentiable Particle-Field Molecular Dynamics

**DOI:** 10.1021/acs.jcim.4c00564

**Published:** 2024-07-04

**Authors:** Manuel Carrer, Henrique Musseli Cezar, Sigbjørn Løland Bore, Morten Ledum, Michele Cascella

**Affiliations:** Hylleraas Centre for Quantum Molecular Sciences and Department of Chemistry, University of Oslo, PO Box 1033, Blindern, 0315 Oslo, Norway

## Abstract

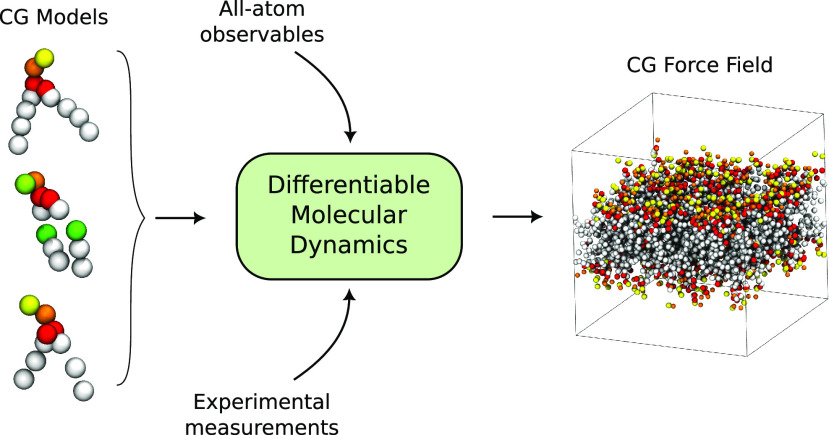

We develop ∂-HylleraasMD (∂-HyMD), a fully end-to-end differentiable molecular dynamics software based on the Hamiltonian hybrid particle-field formalism, and use it to establish a protocol for automated optimization of force field parameters. ∂-HyMD is templated on the recently released HylleraaasMD software, while using the JAX autodiff framework as the main engine for the differentiable dynamics. ∂-HyMD exploits an embarrassingly parallel optimization algorithm by spawning independent simulations, whose trajectories are simultaneously processed by reverse mode automatic differentiation to calculate the gradient of the loss function, which is in turn used for iterative optimization of the force-field parameters. We show that parallel organization facilitates the convergence of the minimization procedure, avoiding the known memory and numerical stability issues of differentiable molecular dynamics approaches. We showcase the effectiveness of our implementation by producing a library of force field parameters for standard phospholipids, with either zwitterionic or anionic heads and with saturated or unsaturated tails. Compared to the all-atom reference, the force field obtained by ∂-HyMD yields better density profiles than the parameters derived from previously utilized gradient-free optimization procedures. Moreover, ∂-HyMD models can predict with good accuracy properties not included in the learning objective, such as lateral pressure profiles, and are transferable to other systems, including triglycerides.

## Introduction

Coarse-grained modeling enables the simulation of systems on length and time scales that are orders of magnitude larger than those accessible in traditional all-atom simulations.^1^ By adopting a low-resolution representation of molecules and computationally efficient interaction potentials, coarse-graining is associated with a smoothening of the molecular free-energy landscape.^2^ Due to the contraction of the phase-space, dynamics of coarse-grained models is intrinsically accelerated, providing additional speedup and faster statistical sampling over all-atom simulations, beyond the increased speed due to lower-resolution molecular representation and fast effective interaction potentials,^3^ even though this comes at the cost of a generally poor description of dynamical properties, such as diffusion coefficients, unless explicitly accounted for.^4^ Acceleration in the conformational sampling enables, for example, the straightforward study of phase separation, like the self-assembly of lipid bilayers,^5,6^ or phase transitions, like spontaneous ice nucleation.^7^

Particle-field models represent the interactions between different molecules through their density fields, leading to a particularly high level of smoothening of the free-energy surface and hence a correspondingly large acceleration of the dynamics.^8^ In fact, we reported that particle-field molecular dynamics models enable (sub)nanosecond equilibration of dispersed charged surfactants to fully self-assembled structures even in the presence of large activation energies^9,10^ that would significantly lengthen the characteristic self-assembling times even using particularly successful CG models like the MARTINI.^11−13^ As such, particle-field approaches provide a powerful tool for studying processes that are computationally inaccessible by standard molecular modeling.

While particle-field simulations are maturing both in terms of mathematical foundations^14,15^ and open-source software implementation,^9,16,17^ the parametrization of the particle-field force field remains challenging. On top of being coarse-grained, which targets the more elusive potential of mean force instead of the potential energy surface of all-atom simulations, the determination of the nonstandard particle-field interactions are not straightforwardly amenable to traditional techniques, such as iterative Boltzmann inversion or force-matching.^18,19^ The key challenge resides in calibrating the -parameter matrix, which describes the mixing energy between the density of two species *k* and *l*. Earlier works investigating polymeric systems typically considered a confined range of χ̃-parameters to understand the nature of their phase diagram.^8^ In subsequent works targeting biological lipids, the χ̃-parameters were chosen by a combination of inference from MARTINI Lennard-Jones parameters and by-hand adjustments in order to reproduce all-atom density profiles.^20^ To systematize the parametrization of χ̃-parameters, some of us proposed an automated machine-learning procedure.^21^ There, similar to the self-driving laboratories approach in the Guzik Group,^22^ Bayesian Optimization (BO) was used to run simulation experiments that compute density profiles for different χ̃-values, determining the optimal χ̃-parameters that maximize the agreement with all-atom reference profiles. The approach is very general and has the advantage over bottom-up coarse-graining approaches in that it can incorporate both experimental and all-atom data, as exemplified in a recent work, where the loss function to parametrize dipalmitoyl-phosphatidylcholine (DPPC) phospholipid bilayers included both the computationally determined density profiles and the experimental value of the area-per-lipid.^23^

BO provides a state-of-the-art algorithm for optimizing costly fitness functions without access to gradients in low-dimensional parameter spaces.^24^ However, this approach becomes problematic when the number of particle species *S* increases, resulting in a large *S*(*S*–1)/2-dimensional parameter space, or, even worse when also including intramolecular bonded parameters into the optimization problem. Optimization techniques employing gradients generally achieve better convergence and work well on higher-dimensional spaces; therefore, they are better suited for force field parametrization. In this regard, differentiable molecular dynamics, leveraging advancements in GPU-based hardware, and highly efficient implementations of automatic differentiation (autodiff),^25^ a generalized back-propagation algorithm^26^ is becoming increasingly popular. Differentiable molecular dynamics can be used to optimize force field parameters through the dynamical evolution of the systems under study. A conventional machine-learning-based approach consists in training machine learning potentials, often based on artificial neural networks (NN), on high-accuracy data (for example, either DFT calculation data sets or all-atom simulations for CG modeling).^27,28^ This methodology has been successfully employed to determine NN pair potentials for CG water models that replicate radial^29,30^ and angular distribution^31^ functions, a NN potential for a CG model of chignolin^32^ and the parametrization of a CG protein force field, where the parameters are trained to minimize the root-mean-square deviation (RMSD) from the native state structures.^33^ However, differentiable molecular dynamics is not limited to training NN potentials; in principle, it can also be applied to the optimization of known analytical functions, like those that are still commonly used in molecular simulations.

Given these successes, in the following, we present ∂-HylleraasMD (∂-HyMD), a reimplementation of the HylleraasMD (HyMD)^9,17^ code for Hamiltonian hybrid particle-field (HhPF) molecular dynamics in the JAX^34,35^ differentiable framework, that allows to perform general force field parametrization on *any* target observable that depends on the simulation trajectory. Our main goal is to optimize the intermolecular interactions described by HhPF. As a test case, we apply our optimization protocol on a diverse set of lipids, providing a library of parameters that can be used for future studies. We also test the performance of these parameters for the self-assembly of lipid bilayers and the transferability to similar systems such as triglycerides.

## Methods

### Hamiltonian Hybrid Particle-Field Dynamics

We start by briefly summarizing the Hamiltonian hybrid particle-field molecular dynamics approach,^9,14^ for which parameters will be optimized. Consider a system of *M* molecules, with the *i*^*th*^ molecule containing *N*_*i*_ particles at positions  with conjugate momenta , subject to the Hamiltonian^14^
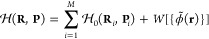
1with
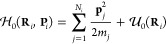
2

Here **R** and **P** denote the collection of positions and momenta of all particles,  is the noninteracting Hamiltonian for the single *i*^*th*^ molecule,  is the intramolecular potential energy (containing only bonded terms) and  is the interaction energy functional, dependent on the densities of the particles {ϕ̃}, modeling the intermolecular interactions. We employ the shorthand  for the collection of number densities associated with all *S* different particle species in the system. This hybrid approach aims to achieve molecular resolution through  and a smooth free energy landscape by adopting a density-dependent interaction energy functional, with minimal steric hindrance. Since  is nothing but a standard molecular mechanics Hamiltonian, in the following we focus on . We adopt a variation of the commonly used Flory–Huggins interaction functional:
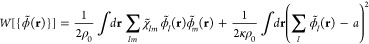
3where ρ_0_ is defined as the density of a coarse-grained particle, κ is the compressibility, which controls the local fluctuations of the density, *a* is a free parameter that can be tuned to calibrate the correct average density at the target temperature and pressure of interest and finally  are the mixing interaction parameters between particle densities of types  and *m*. These model parameters are the optimization variables for our present study.

The sampling of eq 3 can be achieved in multiple ways, such as in Monte Carlo, often referred to as the Single-Chain-In-Mean-Field method,^8^ or by molecular dynamics with various formulations.^9,17,36,37^ We adopt our recently developed Hamiltonian approach, which is the only implementation demonstrated to achieve energy-conserving and alias-free dynamics.^14^ In brief, this approach builds on standard particle-mesh operations^38^ by assigning particle number densities onto a regular grid via an assignment function *P* and subsequently performing a convolution with a filter function  that defines the density spread associated with particle species *t* as
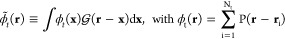
4

The force on a particle placed at **r**_*i*_ is then obtained by the direct spatial derivative of this interaction energy functional as
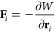
5

The above can be recast, as worked out in ref.,^14^ into the form:
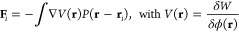
6where *V*(**r**) is called the *external potential*, and its gradients are obtained numerically by Fast Fourier Transform (FFT) operations.^14^

### Differentiable Molecular Dynamics

Our main objective is to minimize an arbitrary loss function , which depends on an observable *A*, with respect to a set of *T* force field parameters ,
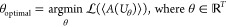
7

Here, the angle brackets denote the ensemble average, while *U*_θ_ = *U*_θ_(**R**; Θ_1_, Θ_2_, ..., Θ_*T*_) is the potential energy depending parametrically on θ. Assuming ergodicity, the ensemble average of the observable *A* is calculated as the time average over a trajectory composed of *N*_*f*_ frames by
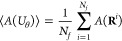
8where superscript *i* denotes positions and velocities of all particles, **R**^*i*^ and **V**^*i*^ respectively, at discrete time step *i*. The particle positions are evolved in time through a discrete and recursive update step:
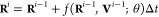
9where Δ*t* the time step and *f* denotes the update scheme. In our implementation, we use the rRESPA integrator,^39^ where the positions-update step is the same as in the velocity-Verlet algorithm. Therefore, we have
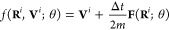
10where **F** = −∇*U*_θ_ are the forces acting on the particles, which directly depend on the force-field parameters. By tracing the update step, we can find the corresponding gradient of the loss function with respect to the force field parameters by recursively applying the chain rule:
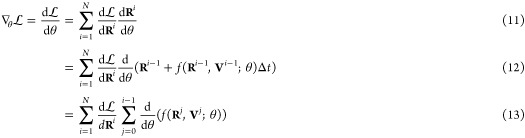
Finally, with this gradient, we can use any gradient descent optimizer to steer the θ-parameters toward the optimum by updating the force field parameters


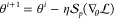
14

where η is the learning rate and  represents the update step function of the given optimizer. Various packages implementing differentiable molecular dynamics are available, such as JAX-MD,^40^ TorchMD,^32^ and Molly.^41,42^ However, these programs lack some critical features, especially the particle mesh routines needed for the HhPF nonbonded interactions. Therefore, we have implemented our differentiable framework in ∂-HyMD based on our open-source HhPF simulator HyMD.^9,17^ In ∂-HyMD we use JAX^34,35^ to trace the update steps and get the gradient of the loss function as in eq 13, while implementing the HhPF operations with fast Fourier transforms, the MD integrator, barostat and thermostat using JAX NumPy API, and taking advantage of JIT compilations whenever possible.

### Differentiable Density Profiles

As a requirement of JAX and the optimizer, the loss function needs to be continuously differentiable. In our case, we want to target the membrane lateral density profile, which is typically obtained by computing the histogram of the *z* coordinate of the particles (assuming here that *z* is the direction normal to the membrane). To calculate a differentiable density profile, we can approximate the histogram with a simple Gaussian kernel density estimation, where we center a normal distribution around each discrete value *z*_*i*_:
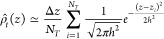
15

Here *N*_*T*_ is the total number of particle of type *T*, Δ*z* is the bin width and *h* is the Gaussian bandwidth. Therefore, the final density profile is given by
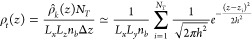
16where *L*_*x*_ and *L*_*y*_ are the box lengths along the respective axis, and *n*_*b*_ is the number of bins. A similar approach can be used if one wants to target, e.g., radial distribution functions, as done in previous works using BO.^43^

### Optimization Protocol

**Figure 1:**
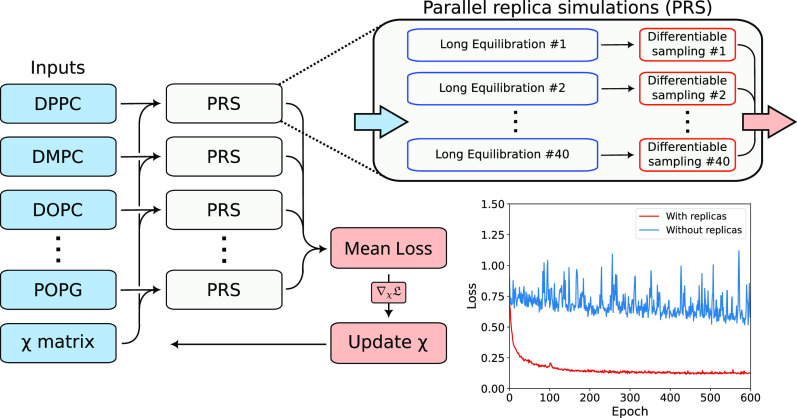
Differentiable MD protocol for the optimization of the lipid library. On the left is the overall training architecture, in which, for each lipid, we run a parallel replica simulation (PRS) without any communication. The results are combined to compute the mean loss (eq 19), which is the quantity effectively minimized. In the zoomed-in part at the top right, a single lipid optimization unit – PRS – which is composed of independent parallel simulations containing an equilibration followed by differentiable sampling. Each PRS unit produces a loss function defined by eq 18. On the bottom right, comparison between the losses obtained from training a single DPPC membrane, with and without replicas.

Differentiable MD has two main drawbacks. First, the memory used in the optimization scales linearly with both the number of particles and the number of simulation steps due to the requirement of tracing all the operations in the MD algorithm. For a single 20 ps differentiable MD simulation of a couple thousand beads, the memory allocated for the backpropagation of the gradients can reach ∼9 GB per task. Second, increasing the number of steps risks the so-called gradient explosion, where gradient sums in eq 13 grow instantly larger, leading to disruptive numerical instability. However, an adequate number of frames is needed to obtain a well-sampled property. To the best of our knowledge, only one work^31^ in the literature suggests an approach, using trajectory reweighing via Boltzmann distributions, that tries to alleviate the second problem. In this work, we opt for a parallel setup, as schematized in Figure 1.

The main idea is to spawn independent replica simulations starting from the same initial CG structure and the same set of starting χ parameters, but with different random number generator seeds. We also tried starting from the final configuration of the previous epoch, but this protocol seemed less robust than starting from the same configuration in each epoch. We call this protocol parallel replica simulations (PRS). By employing the PRS, we are able to scatter the memory usage in different computing nodes while obtaining more reliable gradients. Initially, we equilibrated the system, letting it evolve without tracing any operation. This is followed by a shorter differentiable MD simulation to obtain the target property. Our loss function is then computed as the mean of the loss functions over the replicas:
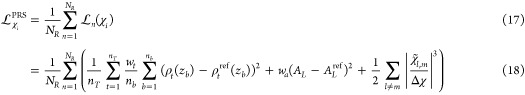
where *N*_*R*_ is the number of replicas. The inner loss function is composed of three terms. The first one targets the density profiles. In this case, *n*_*T*_ is the number of particle types in the system, *n*_*b*_ is the number of bins, *w*_*t*_ is an optional weight parameter, and the densities ρ_*t*_ and ρ_*t*_^ref^ are estimated at the bin centers *z*_*b*_. The second one targets the area per lipid (APL). *A*_*L*_ is the mean APL, naively calculated from the simulations as , *A*_*L*_^ref^ is the experimental value for the APL, and *w*_*A*_ is another optional weight. The last term is a cubic constraint, which prevents the  matrix elements from diverging to unphysical values, avoiding unstable simulations. Here, Δχ is the boundary value.

In Figure 1, we also show the difference between the loss function obtained by training a DPPC membrane with and without the PRS method. Without PRS, the loss is noisy and slowly decreasing, meaning that, due to the short single differentiable MD simulation, we are not able to consistently learn the parameters.

Since training single lipid membranes on their own would lead to nontransferable overfitted parameters to that specific lipid (see Supporting Information), in each epoch, we opt to train a set of them and apply the parameter update step only after all the lipids in the set have been simulated (Figure 1A). This ensures that the parameters are transferable among the different lipids. We achieve this by computing a mean loss
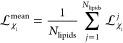
19where  is the PRS loss for the *j*-th of the *N*_lipids_ optimized simultaneously, defined by eq 18. The mean loss is what is effectively minimized and gives the update step for the force field parameters (eq 14).

### Computational Details

All-atom membrane systems for different lipids were set up using CHARMM-GUI,^44^ namely, for 1,2-dipalmitoyl-*sn*-glycero-3-phosphocholine (DPPC), 1,2-dimyristoyl-*sn*-glycero-3-phosphocholine (DMPC), 1,2-dioleoyl-*sn*-glycero-3-phosphocholine (DOPC), 1-palmitoyl-2-oleoyl-glycero-3-phosphocholine (POPC), 1,2-dimyristoyl-*sn*-glycero-3-phosphoethanolamine (DMPE), 1,2-dioleoyl-*sn*-glycero-3-phosphoethanolamine (DOPE), 1-palmitoyl-2-oleoyl-glycero-3-phosphoethanolamine (POPE), 1,2-dipalmitoyl-*sn*-glycero-3-phosphoglycerol (DPPG), 1,2-dimyristoyl-*sn*-glycero-3-phosphoglycerol (DMPG), 1,2-dioleoyl-*sn*-glycero-3-phosphoglycerol (DOPG), and 1-palmitoyl-2-oleoyl-glycero-3-phosphoglycerol (POPG). The box size was approximately 5 × 5 × 8 nm^3^ and the total number of lipid molecules was ∼80, depending on the system. After equilibration, all atomistic MD simulations were run for 100 ns, using a time step of 2 fs, with the CHARMM-36m^45^ force field in GROMACS 2021.5.^46,47^ Temperature control above the melting temperature *T*_*m*_ of each lipid was ensured by using the CSVR^48^ thermostat, with τ_*T*_ = 1 ps, and the pressure was maintained at 1 bar using the semi-isotropic cell rescale barostat,^49^ with τ_*P*_ = 5 ps. PME was used to compute long-range electrostatics, with a real space cutoff radius of 1.2 nm. The same cutoff was also used for the Lennard-Jones interactions, and bonds involving hydrogen atoms were constrained with LINCS.^50^

**Figure 2:**
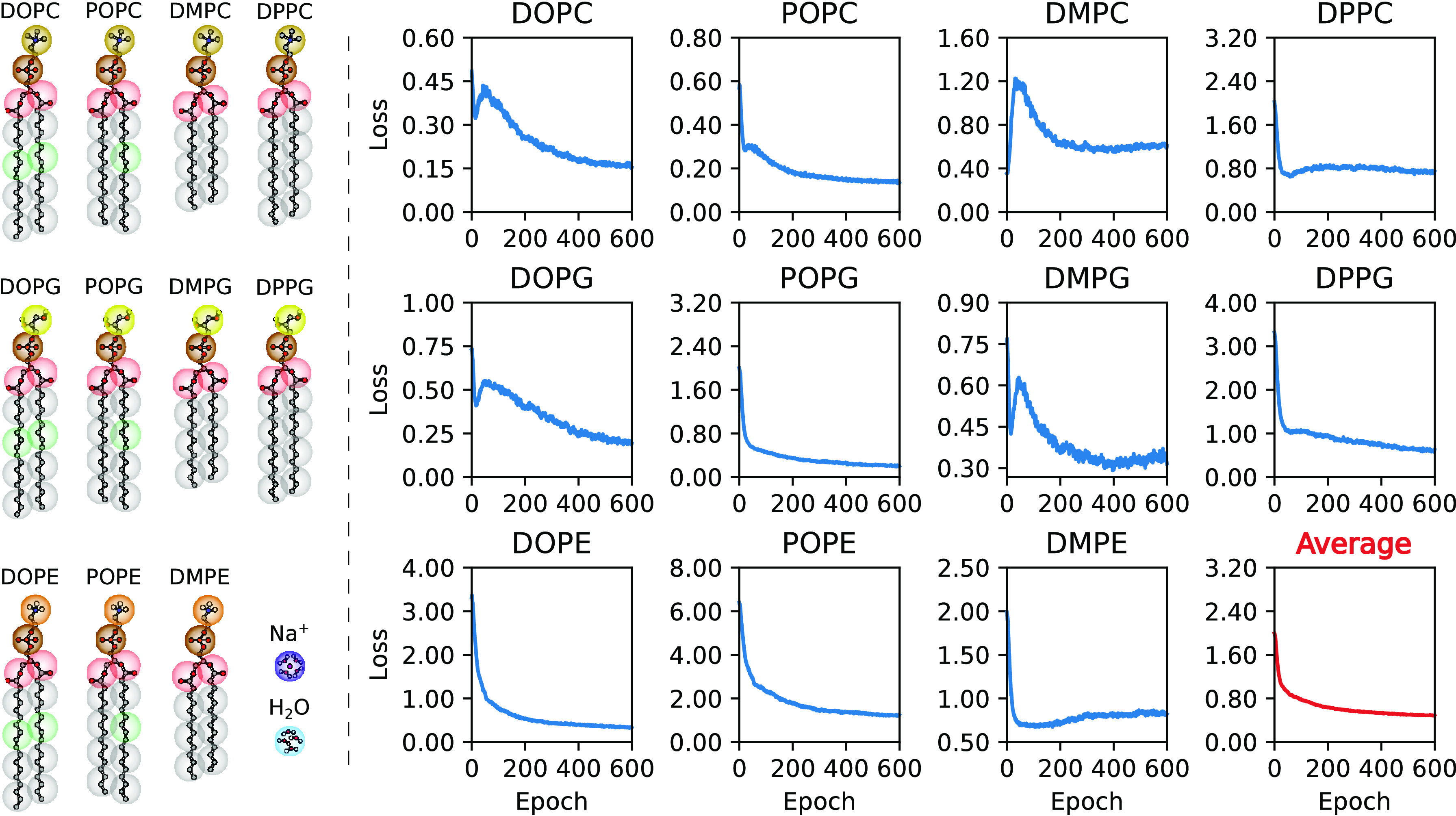
On the left, all-atom to coarse-grained bead mapping. The different bead colors represent the particle types for which the intermolecular interactions were trained in this work. The phosphate groups (represented in brown) are negatively charged (−1), the PC and PE head groups and the Na^+^ ions have positive charge (+1). Apart from the PG and PE head groups, hydrogen atoms were omitted in the all-atom representation. On the right, training loss evolution for the set of phospholipids. Individual losses of each PRS (eq 18) are shown. The panel “Average” contains the mean loss across all the systems, which is the quantity minimized during the optimization procedure (eq 19).

The all-atom trajectories were then coarse-grained using the PyCGTOOL package^51^ and the MARTINI 2 mapping.^52^ The mapping and bead types used for the parametrization are shown in Figure 2, where beads represented by the same color in different lipids have the same χ parameters. The bond distances were calculated from the average distances in the atomistic trajectories (see Table S2 in the Supporting Information), while the reference angles for the three-body interatomic potential and all harmonic force constants were taken from MARTINI. We computed the reference lateral density profiles with MDAnalysis^53,54^ from these CG-mapped trajectories, and used them in the differentiable MD optimization. For what concerns the parallel replica CG simulations, the equilibration was run for 2000 steps (200 ps) while the differentiable MD for 200 steps (20 ps). Since we use 40 replicas for each optimization, the gradients are effectively computed from 40 × 20 ps = 800 ps long simulations. In both equilibration and production, we used a time step of 0.02 ps for the interatomic forces calculation and a time step of 0.1 ps for the intermolecular and electrostatic forces. The systems were kept at constant temperature with the velocity rescale thermostat^48^ and at constant pressure with the semi-isotropic Berendsen barostat.^55^ The coupling constant was set to 0.1 ps for both. We used a 20 × 20 × 30 grid for the particle mesh calculations, compressibility κ = 0.05 kJ^–1^ mol, particle spread σ = 0.5 nm, ρ_0_ = 8.33 nm^–3^ and *a* = 9.21 nm^–3^. The bonded parameters used for the hPF model are reported in Table S2 of the Supporting Information.

Training was carried out over 600 epochs. We set *w*_*t*_ = 1 nm^6^, *w*_*A*_ = 100 nm^–4^, and Δχ = 300 kJ mol^–1^, while for the χ parameter updates, we used the AdaBelief optimizer^56^ with *b*_1_ = 0.1, *b*_2_ = 0.4, and learning rate η = 0.01. However, our implementation allows selecting any optimizer implemented in JAX.

To validate the trained parameters, we employed large unit cells (10 × 10 × 10 *nm*^3^, ∼ 380 lipids) for all the systems. For these systems, we used a 40 × 40 × 40 grid, while keeping all the other simulation parameters unchanged, and ran the simulations for 50 ns. For the DPPC self-assembly validation, we started from the big simulation box system and dispersed the lipids by running a simulation at high temperature, 500 K. Then, to obtain the self-assembled membrane, we ran an NVT simulation at 323 K. Finally, we investigated the phase separation of triolein, a triglyceride (TG), in a DOPC bilayer. The CG mapping of triolein was taken from ref.^57^ We used one of the setups provided in ref.,^58^ where the membrane is composed of 3200 DOPC molecules and 320 TGs. In this case, the box is approximately 33 × 33 × 17 nm^3^ big, and contains ∼113,000 water beads.

## Results

We use ∂-HyMD and the outlined optimization protocol to obtain the  matrix for the nonbonded interaction between each bead in the phospholipid set. The simulations were carried out at temperatures in which the lipids are in the fluid lamellar phase, and experimental data for the APL are available in the literature. The temperatures and experimental APLs are shown in Table 1.

**Table 1:** Reference Experimental Area Per Lipid Compared to the Ones Obtained from Training and from 50 ns Long HhPD MD Simulations of a Larger Membrane Using the Learned  Parameters

System	Experimental (nm^2^)	Training (nm^2^)	Validation (nm^2^)
DPPC	0.633 (50 °C)^60^	0.667 ± 0.004	0.664 ± 0.002
DMPC	0.654 (50 °C)^60^	0.618 ± 0.004	0.612 ± 0.002
DOPC	0.674 (30 °C)^63^	0.669 ± 0.004	0.664 ± 0.002
POPC	0.673 (50 °C)^61^	0.674 ± 0.004	0.667 ± 0.002
DMPE	0.586 (60 °C)^64^	0.553 ± 0.004	0.550 ± 0.002
DOPE	0.600 (22.5 °C)^65^	0.607 ± 0.003	0.603 ± 0.002
POPE	0.566 (30 °C)^66^	0.610 ± 0.003	0.608 ± 0.002
DPPG	0.670 (50 °C)^67^	0.707 ± 0.004	0.702 ± 0.002
DMPG	0.684 (50 °C)^67^	0.660 ± 0.004	0.653 ± 0.002
DOPG	0.729 (50 °C)^67^	0.714 ± 0.004	0.708 ± 0.002
POPG	0.695 (50 °C)^67^	0.713 ± 0.004	0.708 ± 0.002

We optimized the parameters for the set of lipids over 600 epochs, as shown in Figure 2. The mean loss quickly decays during the first 100 steps and keeps going down during the 600 epochs. For most systems, the individual loss value decays in the first 400 epochs and then oscillates around a minimum, usually well below 1. For DMPC, we can see that the loss increases in the first 100 epochs, and only then descends, indicating that the starting parameters were already good for this particular system. However, these parameters were not transferable to other systems, especially to the PG and PE series, which show starting losses above 1.

**Figure 3:**
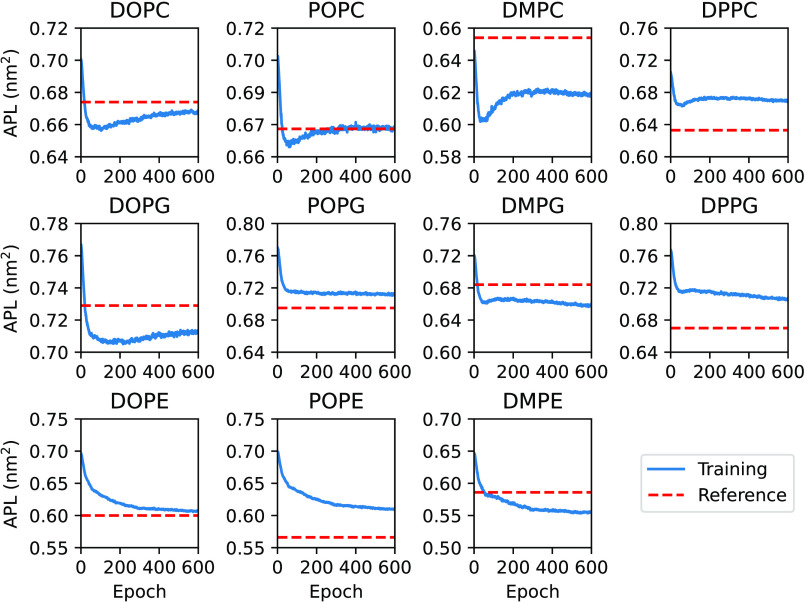
Area per lipid evolution for the set of phospholipids. The horizontal red dashed lines represent the target APL for the system.

The APLs evolution in Figure 3 shows how areas converge toward the target experimental APLs. These curves are, in most cases, highly correlated to the loss evolution curves due to the square difference penalty in the loss function shown in eq 18. For some systems (DOPC, POPC, DOPG, POPG, DMPG, DOPE, DMPE) we obtain very good agreements with the experimental values, while in other cases (DMPC, DPPC, DPPG, POPE) the APL curves seem to diverge. Generally, the mean absolute error is 0.02 nm^2^, corresponding to an average 3.8% deviation from the experimental reference. In the worst case (POPE), the absolute error is around 0.04 nm^2^, (≈7% deviation). It should be noted that these differences are within the variability found for different experiments performed with the same lipid at similar conditions.^59−61^ The average APL deviation is also just slightly larger than the values obtained in a recent refinement of the MARTINI 3 force field, which are up to 4% in their worst cases.^62^ Even though the study of Empereur-mot et al.^62^ also targeted the APL as one of the observables, the authors used different mappings and focused on optimizing the intramolecular bonded terms, which were not the focus of our optimization.

**Figure 4:**
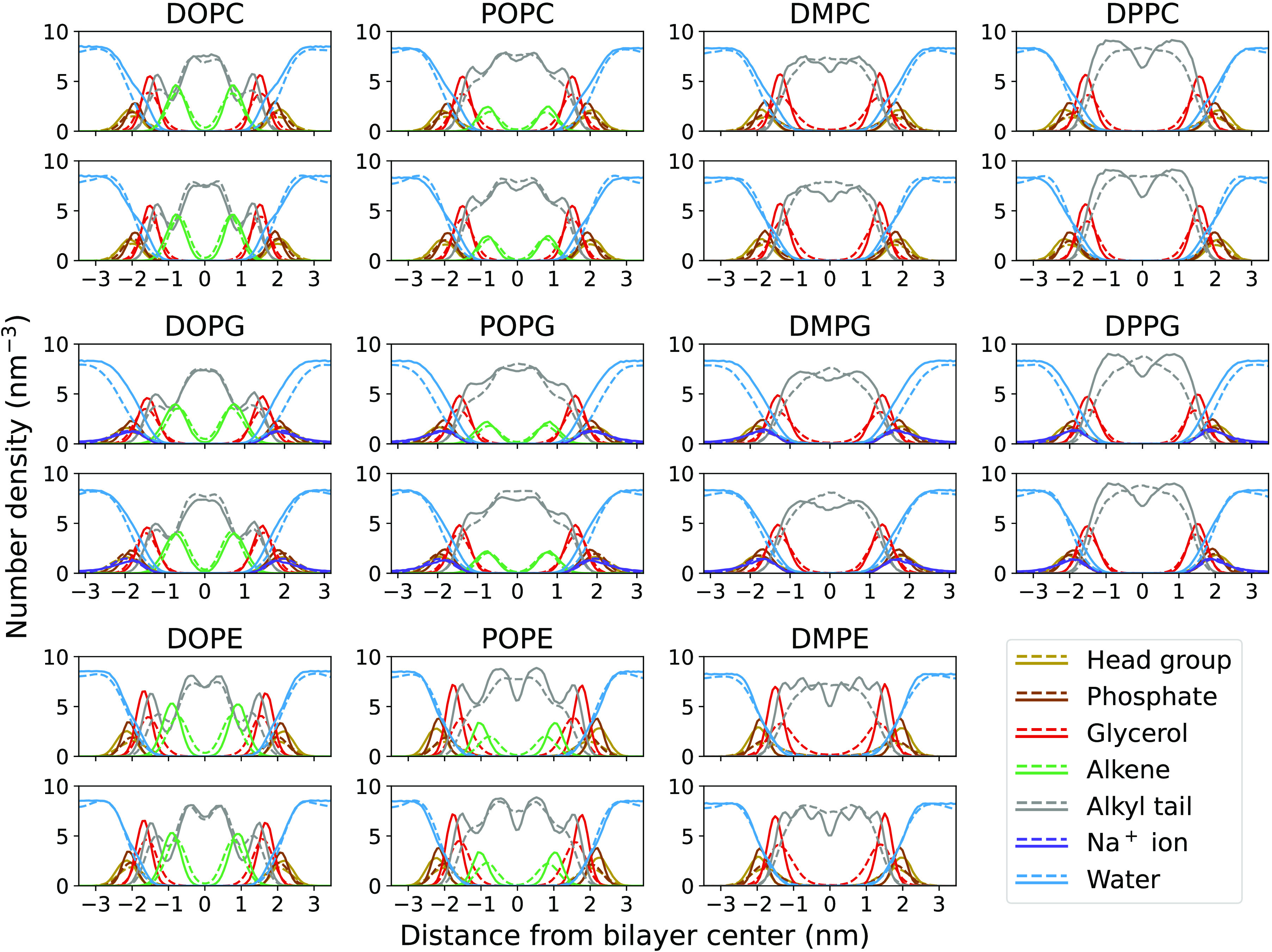
Density profiles for all the optimized lipids (dashed lines), at the start (top) and end (bottom) of training. Solid lines represent the all-atom reference profiles. The same color is used for the head groups.

Despite the loss curves being modulated by the APL, the density profiles also play an important role during the optimization. The density profiles computed in epoch 0, i.e., using the initial set of parameters, and epoch 600 are shown in Figure 4.

We observe a systematic improvement of the density profiles for all the phospholipids, being able to reproduce in most cases peak positions, water penetration, and even structuring of the alkyl tails as seen, e.g., for DOPC, DOPE, and POPE. The robustness of these results that use finite sampling time and simulation size is supported by the nearly identical density profiles Figure S1 of the Supporting Information for four times as large systems simulated over 50 ns. For the DM and DP series, even with the improvements brought by the optimization, we observe that the intricacies of the alkyl density profile were not perfectly reproduced. We attribute this to the fixed topology adopted during the optimization, with bond and angle parameters not being optimized. As shown in previous studies, optimizing the bonded parameters can greatly improve the description of coarse-grained potentials.^62^ However, in this work, we focus on optimizing transferable nonbonded parameters (which were kept constant in ref.^62^) and leave refinements of topology and bonded parameters to future work.

Outside the differentiable training simulations, good agreement is also maintained between the converged APL and the experimental values, as reported in Table 1. In all cases, the error is around 0.03 nm^2^. Since the APL was computed naively by considering only the *xy* plane area, thus not considering membrane undulations, our values might underestimate the real APL. Therefore, values below the experimental values may be closer to the reference than they appear. These combined results indicate that the optimized parameters are transferable to larger systems and can be used for production runs.

As a necessary check for the validation of the data sets produced by our optimization setup, we computed the lateral pressure profiles Δ*P*(*z*) for the optimized lipid bilayers, defined as
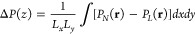
20

**Figure 5:**
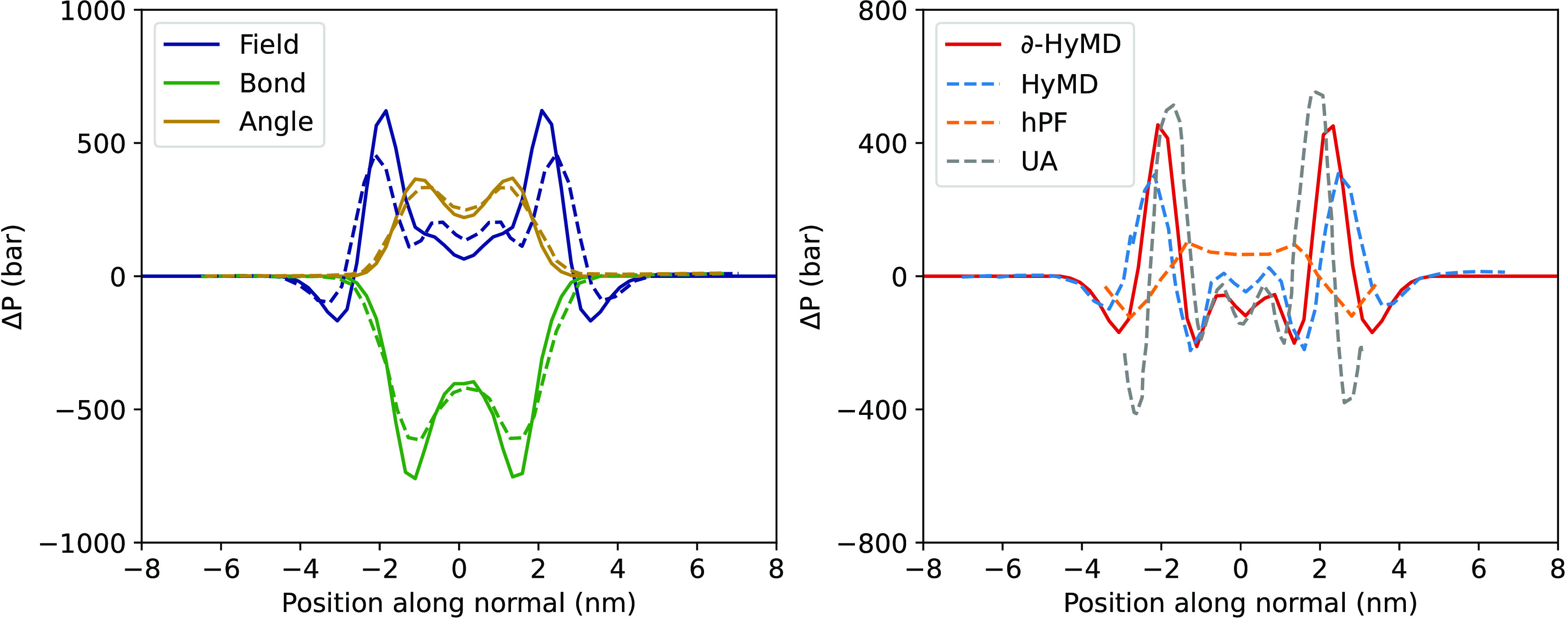
On the left, contributions to the pressure difference in the normal and lateral directions to the membrane (Δ*P*), across a DPPC bilayer simulated with the parameters optimized in this paper (solid lines) and with previous BO parameters from Sen et al.^23^ (dashed lines) due to field, bond and angle terms. On the right, total pressure differences calculated in this work (solid lines), and HhPF, hPF, and united atom (UA) curves are taken from ref^23^

As previously shown using BO-optimized parameters for DPPC in HhPF simulations, the main features of Δ*P*(*z*) could be reproduced without directly introducing any related information in the learning function.^23^Figure 5 reports the lateral pressure profile for the same lipid obtained from simulations using parameters by ∂-HyMD, also in comparison with previously reported data.

Using DPPC for direct comparison, the lateral pressure profile is in excellent quantitative agreement with the one produced by all-atom data, improving the profiles obtained with the BO-optimized parameters. In particular, the lateral pressure profiles feature a weakly negative balance in the membrane core and a large positive fluctuation at the height of the polar head. Notably, in the same region, the stretching, and bending terms provide larger contributions to the pressure imbalance than with the BO model.^23^ This is consistent with the sharper distribution of the polar heads reported in the density profiles (Figure 4), corresponding to a more regular alignment of the bonded moieties along the normal axis of the membrane. Compared to the all-atom model, the pressure profile underestimates the negative fluctuations in the outer part of the bilayer, similar to the BO model. As already discussed there, this is attributed to the poor representation of the solvation structure, which is an intrinsic weakness of the CG mapping itself. However, compared to the BO model, in this case, we managed to almost perfectly match the inner all-atom peaks, both in terms of intensity and position along the normal to the membrane. The qualitatively correct behavior of the lateral pressure profile across the whole membrane validates the soundness of the physics represented by the models and, thus, their reliability for future use in other application studies. Figure S2 in the Supporting Information reports the complete table of lateral pressure profiles for all the other systems.

**Figure 6:**
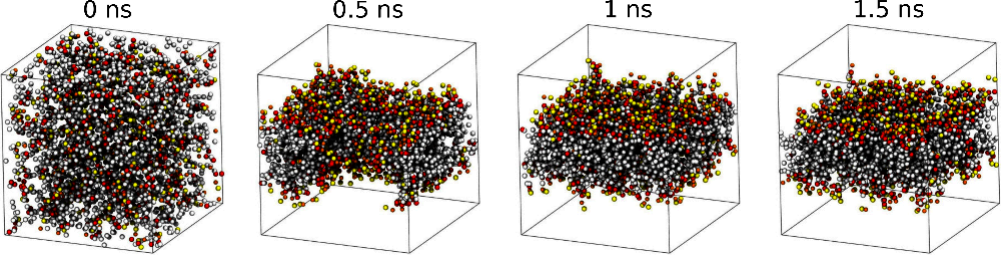
DPPC bilayer self-assembly with the optimized parameters in a 10^3^ nm^3^ box. We use the same color coding shown in the mapping. Water beads are omitted for clarity.

Another validation we carry out is the ability of lipids to self-assemble. This was already investigated in a previous paper,^9^ but it is an important test to replicate here, since the optimized parameters are strikingly different compared to the ones used previously. In particular, the tail bead-water interaction, which is one of the main promoters of lipid aggregation, dropped from 42.24 kJ mol^–1^ to 23.94 kJ mol^–1^. In Figure 6, we report simulation snapshots that show how the new model still manages to reproduce extremely fast self-assembly of a DPPC bilayer, as characteristic of the HhPF model.

**Figure 7:**
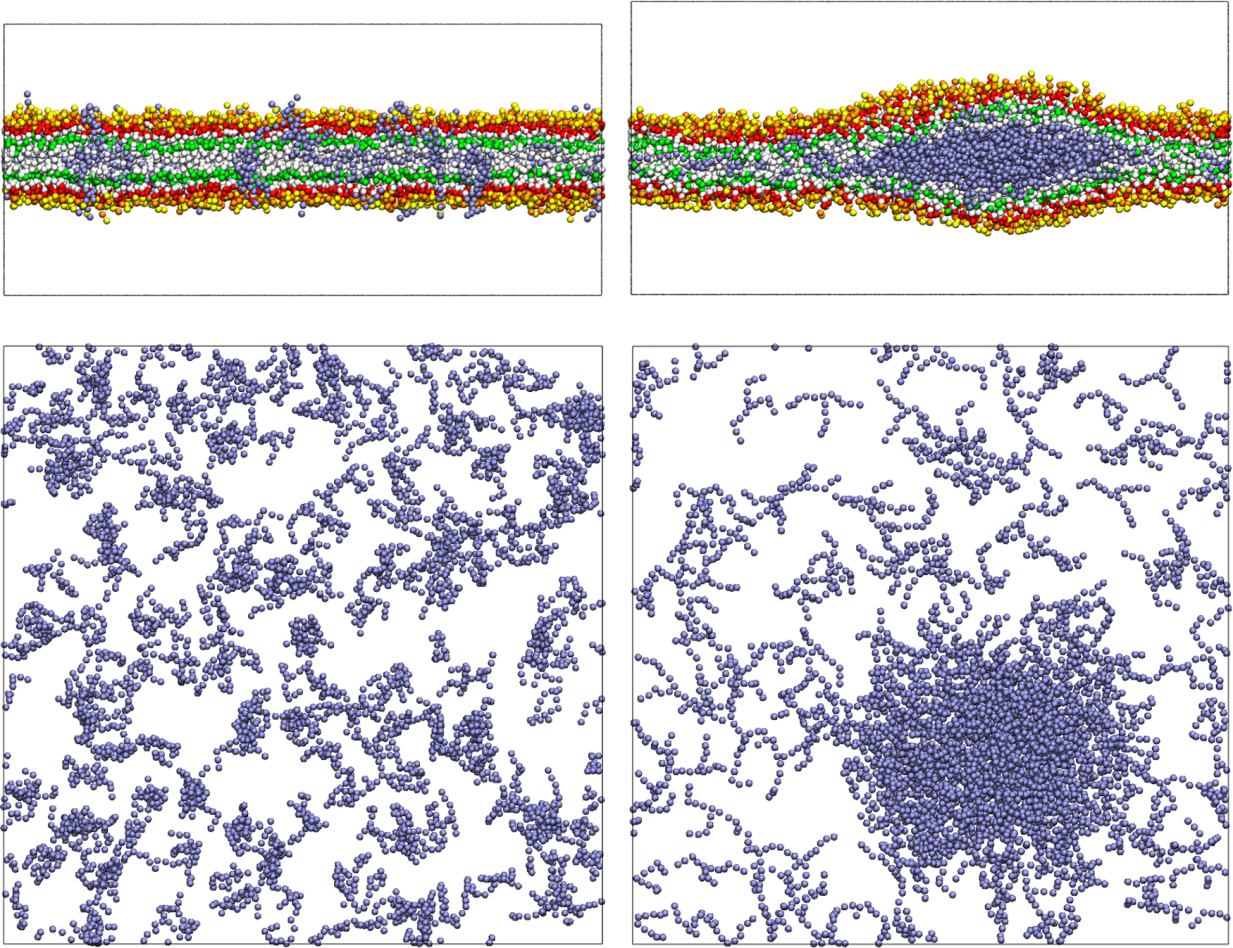
Phase separation of triglyceride (TG) molecules, in blue, inside a DOPC bilayer. On the left, side and top view of the starting system with TGs randomly dispersed in the membrane; on the right, snapshots taken after 30 ns that show the formation of a TG blister. DOPC molecules are not rendered in the top view, and water beads are omitted for clarity.

Finally, we test the phase separation of TG molecules in a DOPC bilayer, that was previously investigated by molecular dynamics simulations and experiments.^58^ We use TG here because the mapping does not introduce any new bead type, so we can also verify the transferability of the optimization on molecules that were not included in the training data set. In our simulation, we use a concentration of TG that is above the reported critical aggregation concentration of 4%,^68^ therefore we expect to observe phase separation from DOPC. In Figure 7 we report snapshots from the initial configuration and after 30 ns. We are indeed able to observe the formation of a TG blister, with a diameter of ∼18 nm and a height of ∼5.7 nm, inside the DOPC membrane. In our simulation, the process of aggregation of the TG molecules starts around the 10 ns mark earlier, but is anyway consistent with the previously reported blister formation time of ∼25 ns.^58^

Overall, the parameters optimized for the whole set of lipids (Table S1 in the Supporting Information) perform just slightly worse than the parameters optimized for each lipid individually (Tables S3 to S14 in the Supporting Information). The data for the losses, density profiles, area per lipid, and lateral pressure profiles for the individual lipids optimization are also shown in Figure S3 to S8 in the Supporting Information. Even though the APL and density profiles for the individually optimized lipids are closer to their experimental references, the parameters were also more prone to overfitting, leading to unstable simulations in some cases. In many cases, the parameters were also very different, so obtaining a single set of transferable parameters from this data is not trivial. Training a common set of parameters for all the lipids with the protocol presented in this work, provided transferable parameters which still lead to properties in very good agreement with the target values.

## Conclusion and Outlook

In this work, we developed and used differentiable molecular dynamics to automate and standardize the determination of force field parameters for HhPF modeling. Our procedure requires only the definition of a loss function, targeting specific molecular properties of interest, which may be based on any microscopic or macroscopic observable, and may target bottom-up data, i.e., from all-atom models, or top-down quantities, i.e., from experimental measurements.

Compared to similar approaches already proposed in the literature, we overcome the general issues of excessive memory requirements, and numerical instability upon progressive gradient accumulation, by averaging the loss gradient over a set of independent parallel runs. This framework is implemented in ∂-HyMD, an open-source software that reimplements HyMD by employing the autodiff capabilities of JAX.

We tested our implementation by optimizing HhPF models for a library of phospholipids, comprising different polar heads (phosphocholine, phosphoethylamine, phosphoglycerol), as well as different combinations of saturated and unsaturated fatty tails. The results show that, in general, our differentiable MD protocol can systematically optimize the parameter sets, producing models that are in excellent agreement with the reference. In particular, the model produced by differentiable MD for DPPC surpasses the one previously obtained by BO, showing better-peaked distributions for the lipid heads, and improved water penetration at the water/lipid interface, even though the parameters are optimized to be transferable to other lipids in the ∂-HyMD case and were tailored to just DPPC in the BO case. These considerations indicate differentiable MD as a more solid route for the systematic optimization of libraries of compounds.

The parameters obtained by ∂-HyMD reproduce with good accuracy the fluctuations in the difference between the normal and lateral components of the pressure, as compared to the all-atom reference, and previous gradient-free parametrization. In particular, they yield the tensionless condition for the bilater as a balance between the laterally compacting hydrophobic tails, and the laterally expanding hydrophilic heads. The good quality of the pressure profiles confirms that the parameters obtained by this optimization procedure describe well the physics of the system, and can be used to predict properties not explicitly introduced into the learning pool. We also showed that the fast self-assembly of lipid membranes is retained by these parameters, and that the extension of their use to molecules outside the training pool, such as triglycerides, is possible.

In this work, we also introduced the first library of standard charged and zwitterionic phospholipids to be used with the HhPF method. In fact, our ∂-HyMD is, by design, a flexible tool that can be easily applied to diverse systems. Future work will be focused on the enrichment of this core library with other biologically relevant lipids, including sphingolipids, sterols, and glycosylated systems (phosphorylated inositol phospholipids, lipopolysaccharides). A further step will be in the use of this same approach for the calibration of sequence-specific peptide models,^69^ and of nucleic acids, with the final objective of producing a consistent force-field for simulations of complex multiphase biological systems. Changing the mapping and including the optimization of the bonded parameters may also lead to improvements in the description of membranes.^62^

## Data Availability

∂-HyMD is an open source software released under LGPLv3 license, freely downloadable at: https://github.com/Cascella-Group-UiO/Diff-HyMD. The HylleraasMD code is also released under LGPLv3 open source software license, freely downloadable at: https://github.com/Cascella-Group-UiO/HyMD. Input and simulations data will become freely available for download upon publication of the present manuscript, at: https://github.com/Cascella-Group-UiO/Publications.

## Supporting Information Available

Additional plots for density profiles and lateral pressure profiles for all lipids, tables with bonded and the optimized χ parameters, and results for individual lipid optimizations (PDF)
