# Supplementary material for: Learning Force Field Parameters from Differentiable Particle-Field Molecular Dynamics
Source: J Chem Inf Model. 2024 Jul 4;64(14):5510–20. doi: 10.1021/acs.jcim.4c00564 (PMC11267579; doi:10.1021/acs.jcim.4c00564)
Supplement: Supplementary file 1 — ci4c00564_si_001.pdf [file ci4c00564_si_001.pdf]

Supporting Information: Learning Force Field Parameters Using Differentiable Particle-Field Molecular Dynamics

Manuel Carrer, Henrique Musseli Cezar, Sigbjørn Løland Bore, Morten Ledum, and Michele Cascella*

Hylleraas Centre for Quantum Molecular Sciences and Department of Chemistry, University of Oslo, PO Box 1033 Blindern, 0315 Oslo, Norway

E-mail: michele.cascella@kjemi.uio.no

Contents

S1 Lipid dataset additional information **S-4**

Figure S1 - Density profiles calculated from the 50 ns long validation simulations in 10 nm cubic boxes for the lipid dataset. S-4

Figure S2 - Lateral pressure profiles for all the lipids parameterized in the lipid dataset. S-5

Table S1 - χ parameters obtained for the lipid dataset. A dashed line means that the pair was not included in the training. The beads PC, PE and Na^+ have a +1 charge, and P has a −1 charge. S-5

Table S2 - Bonded parameters for the lipids. These parameters were used both for the lipids dataset, and the individual lipid optimizations shown below. We only report the bond equilibrium distances in nm, r_0, since all the other parameters (equilibrium angle, bond and angle strengths) were kept as in the original MARTINI 2 mapping[S1]. S-6

S2 Individual lipids optimization **S-7**

Figure S3 - Training loss evolution for the set of phospholipids (single lipid optimization). S-7

Figure S4 - Area per lipid evolution for the set of phospholipids (single lipid optimization). The horizontal red dashed lines represent the target APL for the system. S-8

Figure S5 - Density profiles for all the optimized lipids (dashed lines – single lipid optimization), at the start (top) and end (bottom) of training. Solid lines represent the all-atom reference profile. Color code: gold = head group, dark orange = phosphate, red = glycerol, green = alkene, silver = alkyl tail, purple = Na^+ ion, blue = water. S-9

Figure S6 - Density profiles calculated from the 10 ns long validation simulations (single lipid optimization). Color code: gold = head group, dark orange = phosphate, red = glycerol, green = alkene, silver = alkyl tail, purple = Na^+ ion, blue = water. S-10

Figure S7 - On the left, contributions to the pressure difference in the normal and lateral directions to the membrane (ΔP), across a DPPC bilayer (single lipid optimization parameters) simulated with the parameters optimized in this paper (solid line) and with previous BO parameters from Sen et al.[S2] (dashed line) due to field, bond and angle terms. On the right, total pressure differences calculated in this work (solid line), and HhPF, hPF, and united atom (UA) curves are taken from ref[S2] (single lipid optimization). S-11
Figure S8 - Lateral pressure profiles for all the lipids parameterized in this work (single lipid optimization). Color code: blue = field pressure, green = bond pressure, gold = angle pressure, red = total pressure. S-12
Table S3 - χ parameters for DMPG. Na^+ has a +1 charge, and P has a −1 charge. . . S-13
Table S4 - χ parameters for DMPE. PE has a +1 charge, and P has a −1 charge. . . S-13
Table S5 - χ parameters for POPC. PC has a +1 charge, and P has a −1 charge. . . S-13
Table S6 - χ parameters for DPPE. PE has a +1 charge, and P has a −1 charge. . . . S-14
Table S7 - χ parameters for DOPG. Na^+ has a +1 charge, and P has a −1 charge. . . S-14
Table S8 - χ parameters for DPPG. Na^+ has a +1 charge, and P has a −1 charge. . . S-14
Table S9 - χ parameters for DPPC. PC has a +1 charge, and P has a −1 charge. . . S-14
Table S10 - χ parameters for POPG. Na^+ has a +1 charge, and P has a −1 charge. . . S-14
Table S11 - χ parameters for POPE. PE has a +1 charge, and P has a −1 charge. . . . S-15
Table S12 - χ parameters for DOPC. PC has a +1 charge, and P has a −1 charge. . . S-15
Table S13 - χ parameters for DMPC. PC has a +1 charge, and P has a −1 charge. . . S-15
Table S14 - χ parameters for DOPE. PE has a +1 charge, and P has a −1 charge. . . S-15

References **S-16**

S1 Lipid dataset additional information

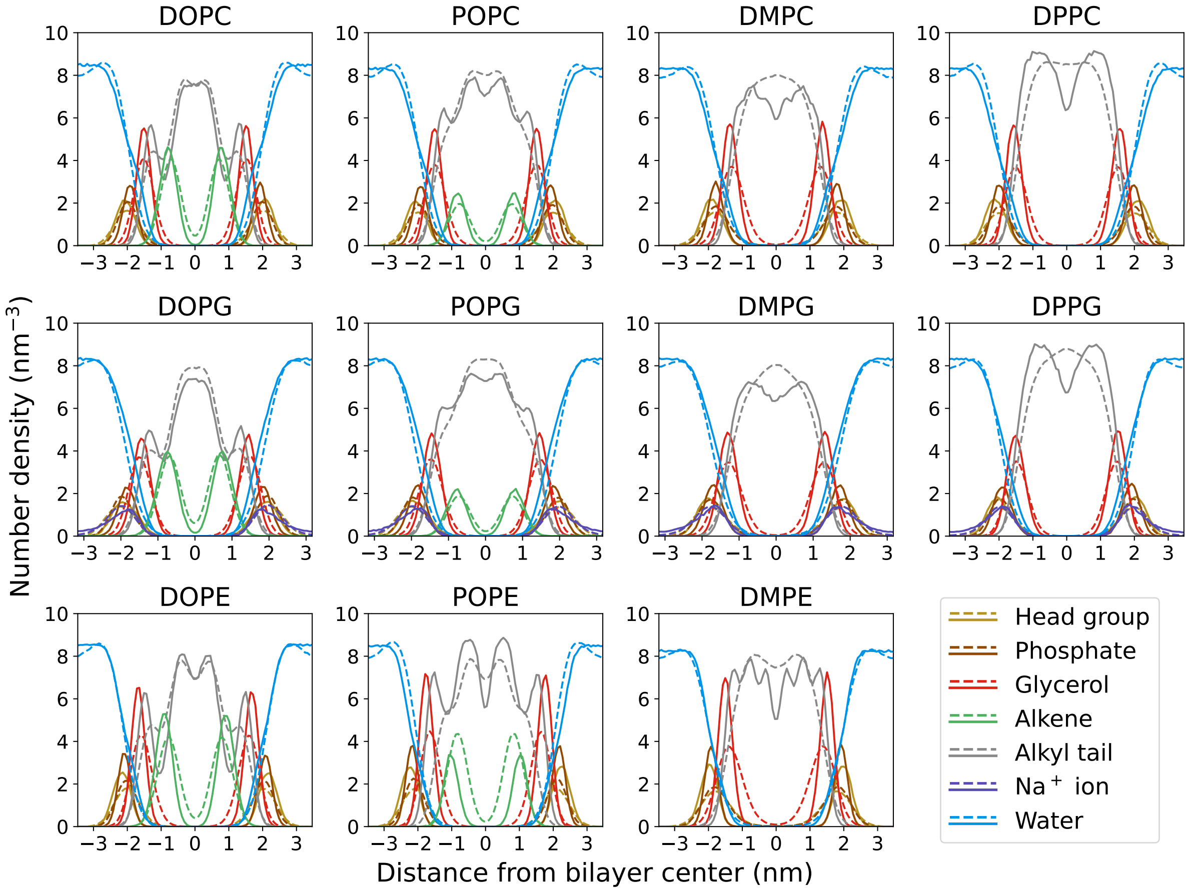

Figure S1: Density profiles calculated from the 50 ns long validation simulations in 10 nm cubic boxes for the lipid dataset.

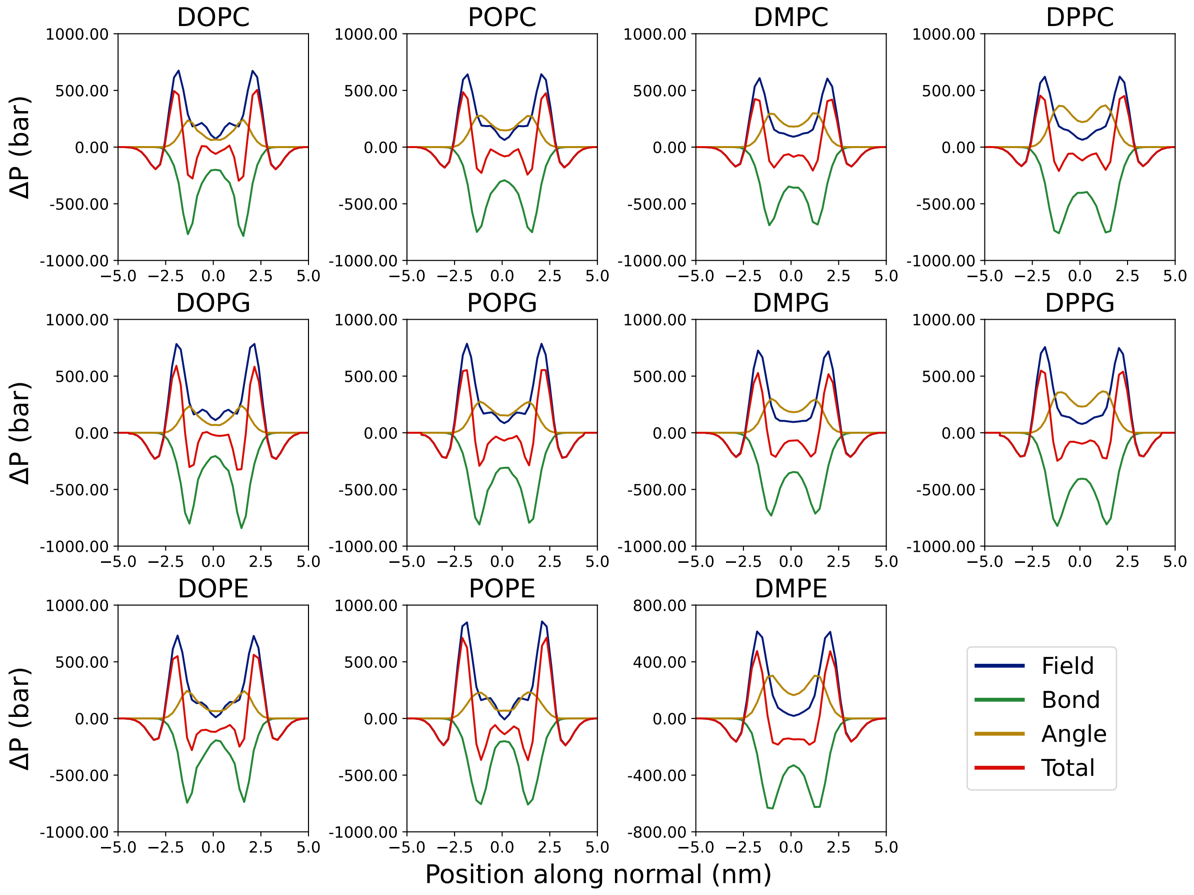

Figure S2: Lateral pressure profiles for all the lipids parameterized in the lipid dataset.

Table S1: χ parameters obtained for the lipid dataset. A dashed line means that the pair was not included in the training. The beads PC, PE and Na^+ have a +1 charge, and P has a -1 charge.

	PC	PE	PG	P	G	C	D	Na^+	W
PC	0.00	—	—	-14.41	-12.53	-9.44	-5.70	—	-22.02
PE		0.00	—	-26.63	-27.94	-16.76	-20.52	—	-18.71
PG			0.00	-19.58	-17.78	-16.86	-10.67	-21.22	-20.03
P				0.00	-30.35	13.68	13.28	-17.44	-12.15
G					0.00	2.48	6.61	-24.44	-21.71
C						0.00	-3.58	12.11	23.94
D							0.00	20.55	19.25
Na^+								0.00	-15.53
W									0.00

Table S2: Bonded parameters for the lipids. These parameters were used both for the lipids dataset, and the individual lipid optimizations shown below. We only report the bond equilibrium distances in nm, r_0, since all the other parameters (equilibrium angle, bond and angle strengths) were kept as in the original MARTINI 2 mapping[S1].

DOPC

atom 1	atom 2	r_0
1	2	0.43
2	3	0.44
3	4	0.33
3	5	0.26
5	6	0.66
6	7	0.41
7	8	0.46
4	9	0.27
9	10	0.67
10	11	0.41
11	12	0.46

POPC

atom 1	atom 2	r_0
1	2	0.43
2	3	0.44
3	4	0.33
3	5	0.26
5	6	0.66
6	7	0.42
7	8	0.46
4	9	0.27
9	10	0.46
10	11	0.47
11	12	0.46

DMPC

atom 1	atom 2	r_0
1	2	0.43
2	3	0.45
3	4	0.33
3	5	0.26
5	6	0.57
6	7	0.57
4	8	0.27
8	9	0.58
9	10	0.57

DPPC

atom 1	atom 2	r_0
1	2	0.43
2	3	0.45
3	4	0.33
3	5	0.26
5	6	0.46
6	7	0.47
7	8	0.46
4	9	0.27
9	10	0.47
10	11	0.47
11	12	0.46

DOPG

atom 1	atom 2	r_0
1	2	0.41
2	3	0.44
3	4	0.33
3	5	0.26
5	6	0.66
6	7	0.41
7	8	0.46
4	9	0.27
9	10	0.67
10	11	0.41
11	12	0.46

POPG

atom 1	atom 2	r_0
1	2	0.41
2	3	0.44
3	4	0.33
3	5	0.26
5	6	0.66
6	7	0.41
7	8	0.46
4	9	0.27
9	10	0.46
10	11	0.46
11	12	0.46

DMPG

atom 1	atom 2	r_0
1	2	0.41
2	3	0.44
3	4	0.33
3	5	0.26
5	6	0.57
6	7	0.57
4	8	0.27
8	9	0.57
9	10	0.57

DPPG

atom 1	atom 2	r_0
1	2	0.41
2	3	0.44
3	4	0.33
3	5	0.26
5	6	0.46
6	7	0.47
7	8	0.46
4	9	0.27
9	10	0.46
10	11	0.47
11	12	0.46

DOPE

atom 1	atom 2	r_0
1	2	0.42
2	3	0.44
3	4	0.33
3	5	0.26
5	6	0.67
6	7	0.42
7	8	0.46
4	9	0.27
9	10	0.68
10	11	0.42
11	12	0.46

POPE

atom 1	atom 2	r_0
1	2	0.42
2	3	0.44
3	4	0.32
3	5	0.26
5	6	0.67
6	7	0.42
7	8	0.47
4	9	0.27
9	10	0.47
10	11	0.48
11	12	0.47

DMPE

atom 1	atom 2	r_0
1	2	0.42
2	3	0.44
3	4	0.33
3	5	0.26
5	6	0.58
6	7	0.58
4	8	0.27
8	9	0.58
9	10	0.58

S2 Individual lipids optimization

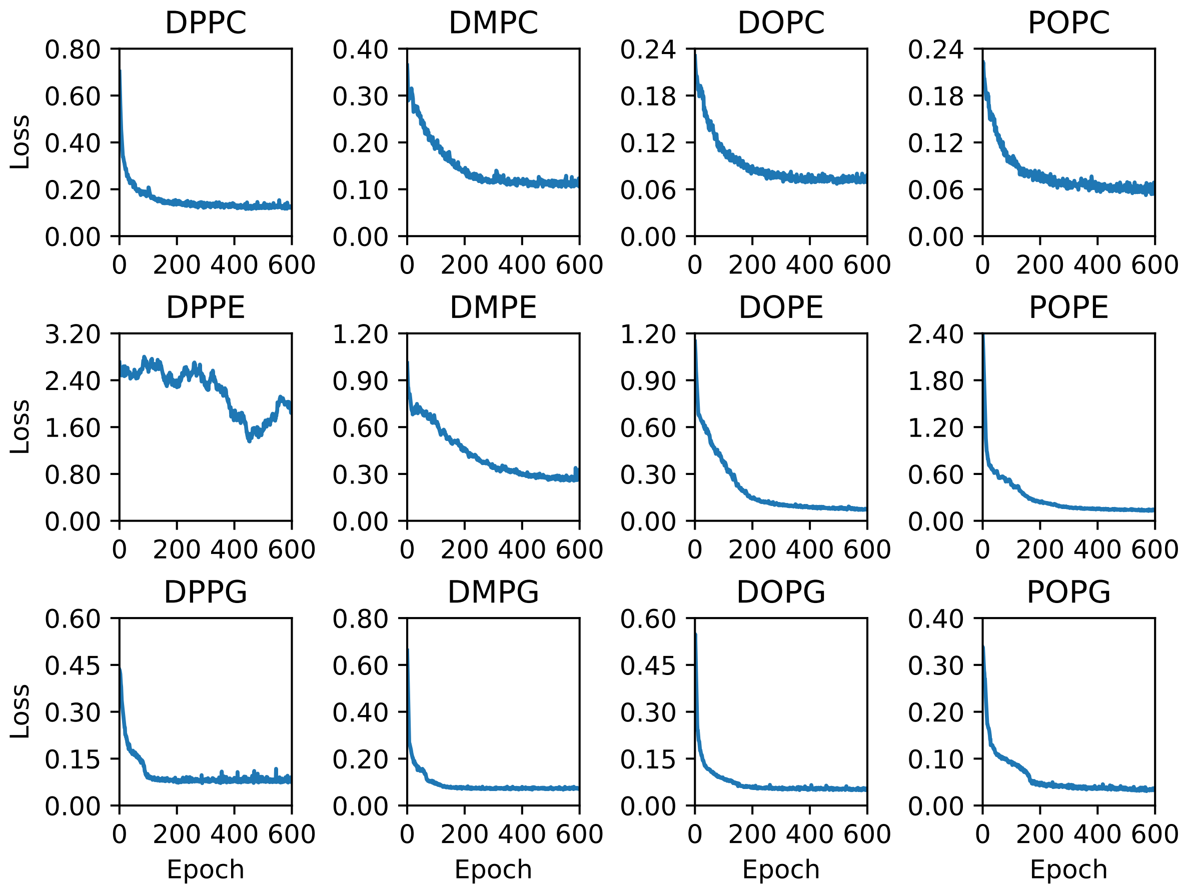

Figure S3: Training loss evolution for the set of phospholipids (single lipid optimization).

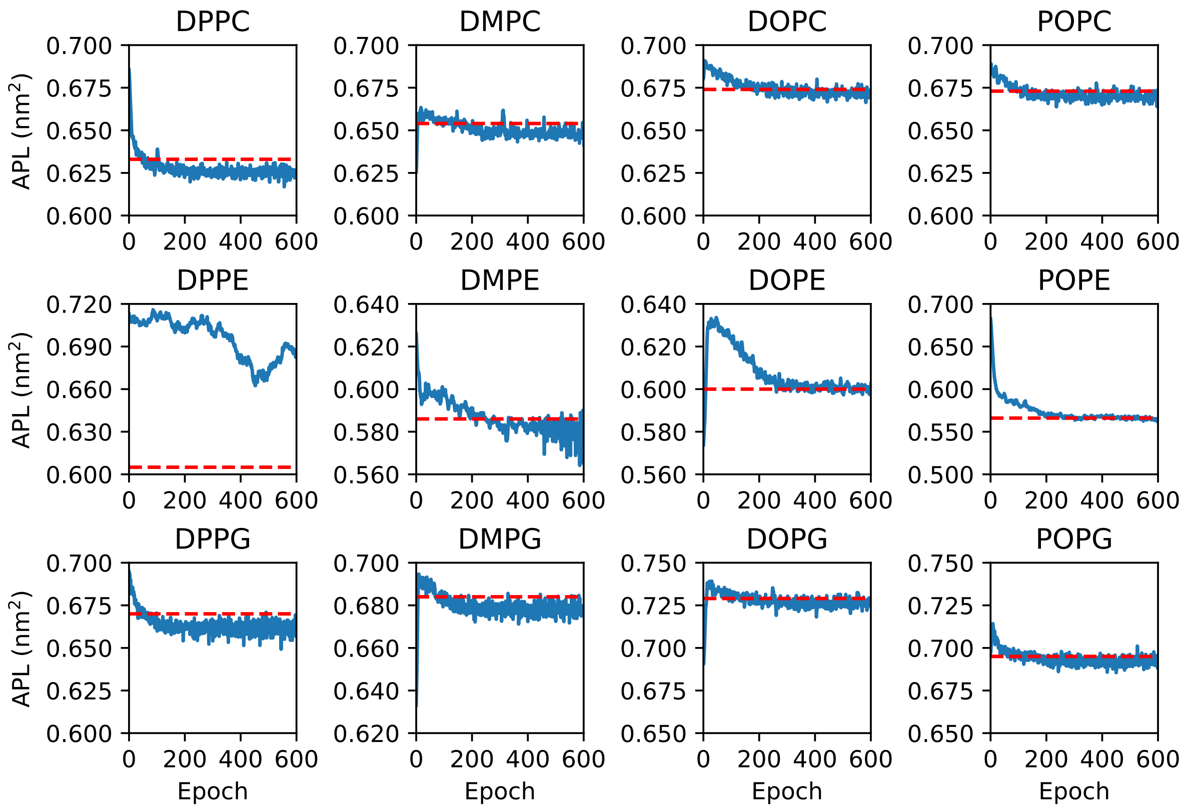

Figure S4: Area per lipid evolution for the set of phospholipids (single lipid optimization). The horizontal red dashed lines represent the target APL for the system.

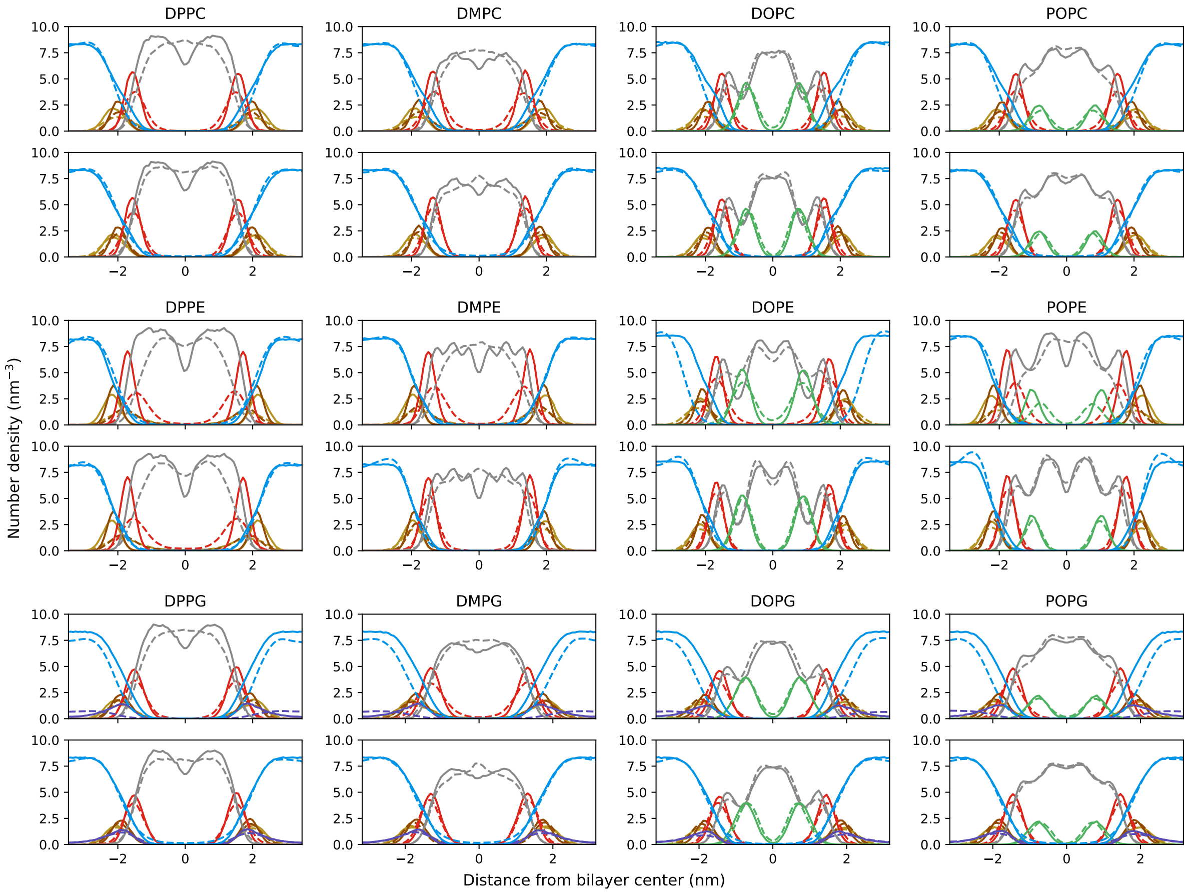

Figure S5: Density profiles for all the optimized lipids (dashed lines – single lipid optimization), at the start (top) and end (bottom) of training. Solid lines represent the all-atom reference profile. Color code: gold = head group, dark orange = phosphate, red = glycerol, green = alkene, silver = alkyl tail, purple = Na^+ ion, blue = water.

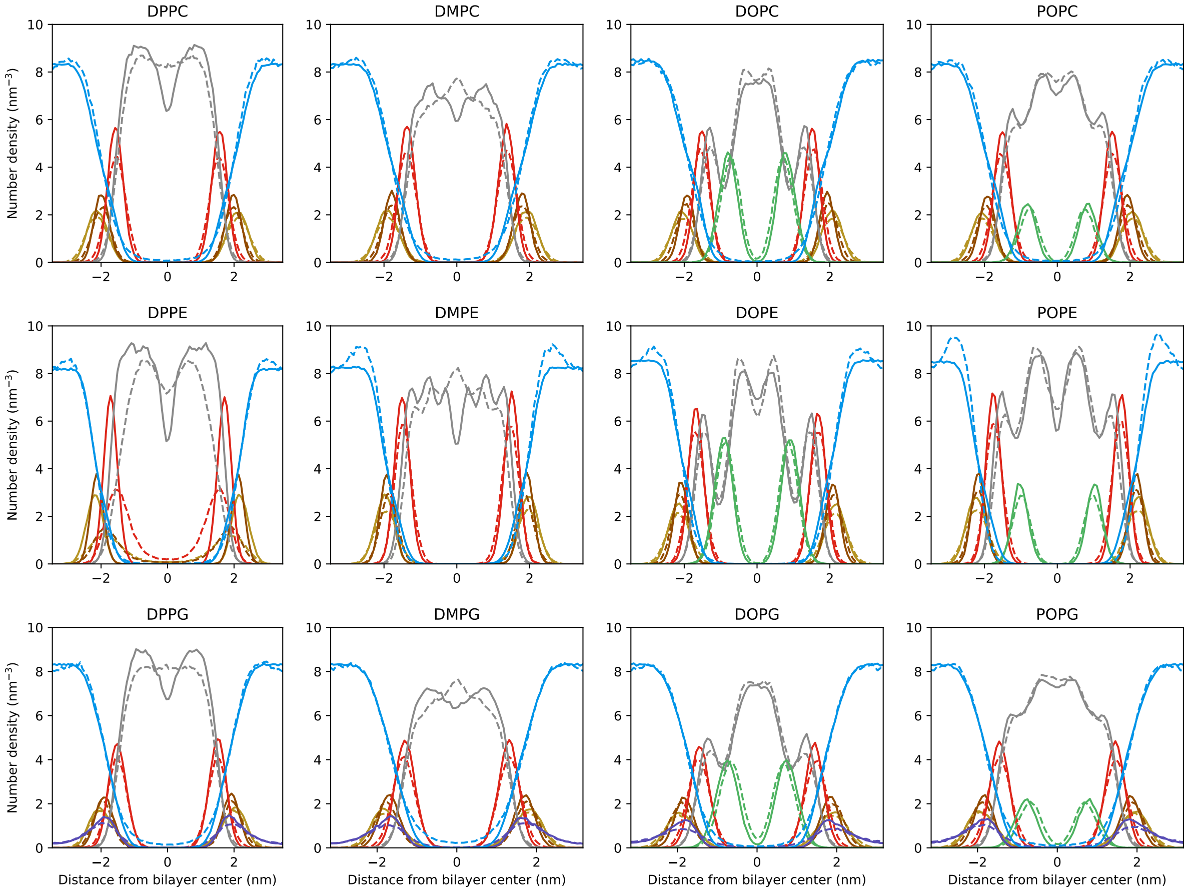

Figure S6: Density profiles calculated from the 10 ns long validation simulations (single lipid optimization). Color code: gold = head group, dark orange = phosphate, red = glycerol, green = alkene, silver = alkyl tail, purple = Na^+ ion, blue = water.

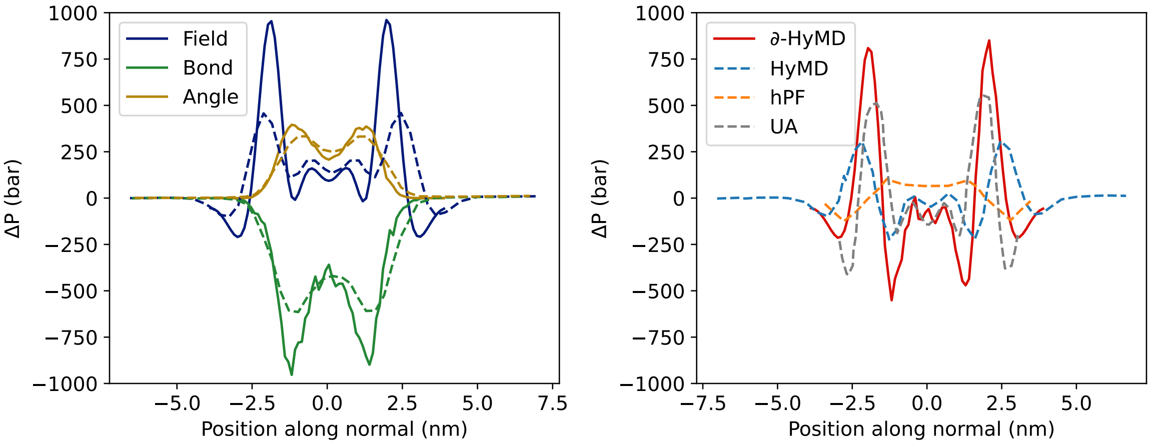

Figure S7: On the left, contributions to the pressure difference in the normal and lateral directions to the membrane (ΔP), across a DPPC bilayer (single lipid optimization parameters) simulated with the parameters optimized in this paper (solid line) and with previous BO parameters from Sen et al.[S2] (dashed line) due to field, bond and angle terms. On the right, total pressure differences calculated in this work (solid line), and HhPF, hPF, and united atom (UA) curves are taken from ref[S2] (single lipid optimization).

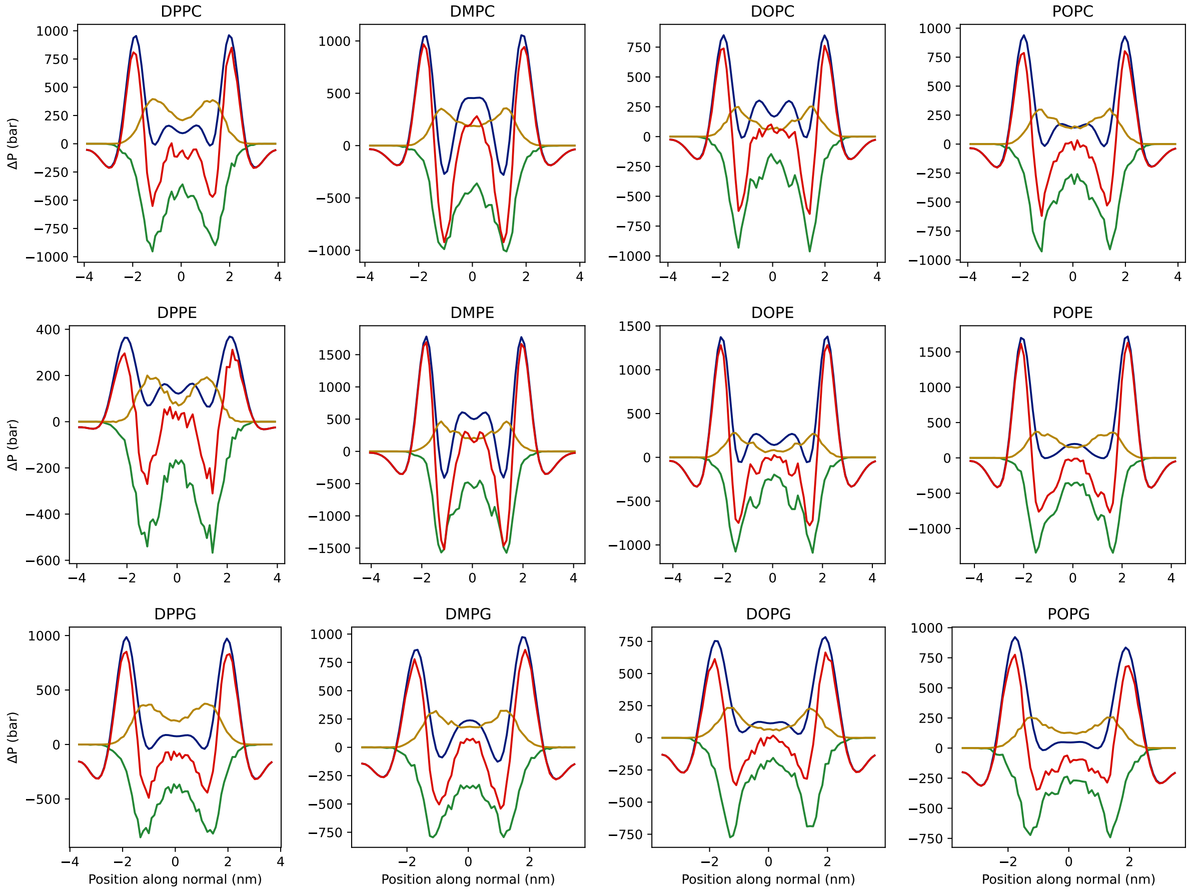

Figure S8: Lateral pressure profiles for all the lipids parameterized in this work (single lipid optimization). Color code: blue = field pressure, green = bond pressure, gold = angle pressure, red = total pressure.

Table S3: χ parameters for DMPG. Na^+ has a +1 charge, and P has a -1 charge.

	PG	P	G	C	Na^+	W
PG		-42.24	-26.16	-14.66	-32.86	-19.78
P			-43.12	18.32	-34.21	-12.45
G				22.47	-61.37	-16.76
C					-2.88	17.72
Na^+						-16.59

Table S4: χ parameters for DMPE. PE has a +1 charge, and P has a -1 charge.

	PE	P	G	C	W
PE		-67.16	-42.5	-7.83	-25.88
P			-83.03	11.01	-7.69
G				47.64	-30.73
C					37.06

Table S5: χ parameters for POPC. PC has a +1 charge, and P has a -1 charge.

	PC	P	G	C	D	W
PC		-52.31	-31.13	-4.68	1.35	-21.14
P			-67.21	24.52	34.25	-3.01
G				7.81	13.91	-16.41
C					3.69	12.37
D						32.75

Table S6: χ parameters for DPPE. PE has a +1 charge, and P has a -1 charge.

	PE	P	G	C	W
PE		-11.92	-10.98	-14.81	-15.15
P			-11.41	18.8	-2.31
G				15.74	-10.68
C					25.14

Table S7: χ parameters for DOPG. Na^+ has a +1 charge, and P has a -1 charge.

	PG	P	G	C	D	Na^+	W
PG		-32.29	-24.18	-16.47	-14.04	-29.15	-18.2
P			-33.45	17.46	24.47	-29.63	-13.66
G				4.83	11.41	-45.52	-18.57
C					-0.05	-12.18	12.95
D						16.51	23.55
Na^+							-13.3

Table S8: χ parameters for DPPG. Na^+ has a +1 charge, and P has a -1 charge.

	PG	P	G	C	Na^+	W
PG		-46.02	-31.63	-11.41	-30.18	-22.03
P			-60.41	17.34	-36.36	-14.23
G				6.38	-65.19	-16.31
C					-3.37	13.21
Na^+						-17.03

Table S9: χ parameters for DPPC. PC has a +1 charge, and P has a -1 charge.

	PC	P	G	C	W
PC		-41.11	-38.08	-0.18	-23.88
P			-91.42	26.42	-4.84
G				6.22	-16.06
C					13.87

Table S10: χ parameters for POPG. Na^+ has a +1 charge, and P has a -1 charge.

	PG	P	G	C	D	Na^+	W
PG		-38.73	-29.67	-15.51	-7.22	-31.6	-21.32
P			-47.11	16.33	33.01	-27.6	-12.39
G				-2.13	22.27	-53.99	-18.88
C					1.19	-9.73	11.16
D						30.07	32.71
Na^+							-14.83

Table S11: χ parameters for POPE. PE has a +1 charge, and P has a -1 charge.

	PE	P	G	C	D	W
PE		-54.76	-41.55	-9.7	-12.02	-23.27
P			-79.31	18.89	17.71	-11.93
G				10.88	-11.99	-39.96
C					15.74	35.36
D						36.69

Table S12: χ parameters for DOPC. PC has a +1 charge, and P has a -1 charge.

	PC	P	G	C	D	W
PC		-50.0	-29.22	-6.34	-5.11	-18.76
P			-61.3	22.26	25.19	-0.76
G				17.44	9.04	-18.15
C					-3.36	14.85
D						13.16

Table S13: χ parameters for DMPC. PC has a +1 charge, and P has a -1 charge.

	PC	P	G	C	W
PC		-56.35	-34.94	-3.23	-22.14
P			-79.81	23.99	-3.71
G				36.59	-14.39
C					19.09

Table S14: χ parameters for DOPE. PE has a +1 charge, and P has a -1 charge.

	PE	P	G	C	D	W
PE		-53.05	-45.48	-11.9	-14.36	-24.23
P			-79.37	20.06	17.41	-17.11
G				28.86	-14.25	-19.18
C					2.5	22.24
D						18.02

References

(S1) Marrink, S. J.; de Vries, A. H.; Mark, A. E. Coarse Grained Model for Semiquantitative Lipid Simulations. J. Phys. Chem. B **2004**, 108, 750–760.

(S2) Sen, S.; Ledum, M.; Bore, S. L.; Cascella, M. Soft Matter under Pressure: Pushing Particle– Field Molecular Dynamics to the Isobaric Ensemble. J. Chem. Inf. Model. **2023**, 63, 2207–2217.
